# Use of Untargeted Liquid Chromatography–Mass Spectrometry Metabolome To Discriminate Italian Monovarietal Red Wines, Produced in Their Different Terroirs

**DOI:** 10.1021/acs.jafc.0c00879

**Published:** 2020-04-09

**Authors:** Panagiotis Arapitsas, Maurizio Ugliano, Matteo Marangon, Paola Piombino, Luca Rolle, Vincenzo Gerbi, Andrea Versari, Fulvio Mattivi

**Affiliations:** † Department of Food Quality and Nutrition, Research and Innovation Centre, Fondazione Edmund Mach (FEM), Via Edmund Mach 1, 38010 San Michele all’Adige, Trentino, Italy; ‡ Department of Biotechnology, University of Verona, Cà Vignal 1, Strada le Grazie 15, 37134 Verona, Italy; § Department of Agronomy, Food, Natural Resources, Animals and Environment (DAFNAE), University of Padua, Viale dell’Università 16, 35020 Legnaro, Padua, Italy; ∥ Department of Agricultural Sciences, Division of Vine and Wine Sciences, University of Naples Federico II, Viale Italia, 83100 Avellino, Italy; ⊥ Department of Agricultural, Forest and Food Sciences, University of Turin, Largo Paolo Braccini 2, 10095 Grugliasco, Turin, Italy; # Department of Agricultural and Food Sciences, University of Bologna, Piazza Goidanich 60, 47521 Cesena, Forlì-Cesena, Italy; ∇ Department of Cellular, Computational and Integrative Biology (CIBIO), University of Trento, 38123 Povo, Trentino, Italy

**Keywords:** mass spectrometry, wine authenticity, biomarkers discovery, wine metabolomics, amines, polyphenols

## Abstract

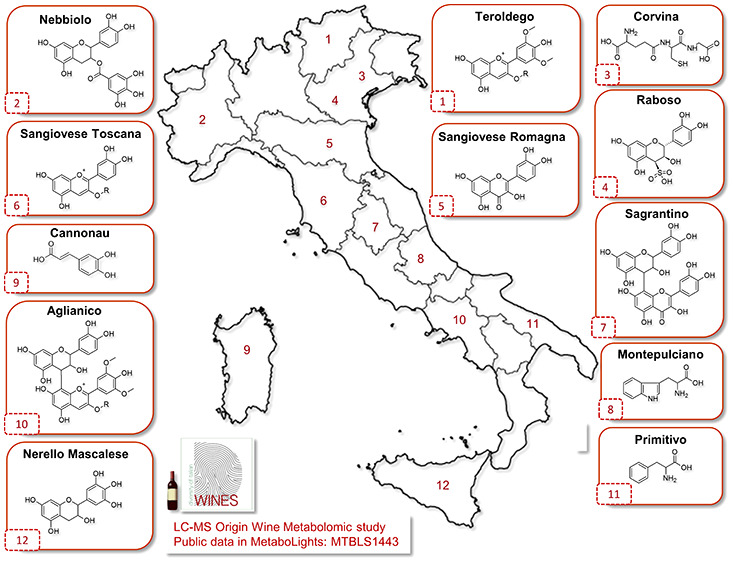

The aim of this project was to register, in a liquid chromatography–mass spectrometry-based untargeted single-batch analysis, the metabolome of 11 single-cultivar, single-vintage Italian red wines (Aglianico, Cannonau, Corvina, Montepulciano, Nebbiolo, Nerello, Primitivo, Raboso, Sagrantino, Sangiovese, and Teroldego) from 12 regions across Italy, each one produced in their terroirs under *ad hoc* legal frameworks to guarantee their quality and origin. The data provided indications regarding the similarity between the cultivars and highlighted a rich list of putative biomarkers of origin wines (pBOWs) characterizing each individual cultivar–terroir combination, where Primitivo, Teroldego, and Nebbiolo had the maximum number of unique pBOWs. The pBOWs included anthocyanins (Teroldego), flavanols (Aglianico, Sangiovese, Nerello, and Nebbiolo), amino acids and N-containing metabolites (Primitivo), hydroxycinnamates (Cannonau), and flavonols (Sangiovese). The raw data generated in this study are publicly available and, therefore, accessible and reusable as a baseline data set for future investigations.

## Introduction

Italy is one of the most important countries in the world with regard to viticulture and oenology, with 705 000 ha of vineyards (4th place), grape production of 8.6 million tons (2nd place), wine production of 54.8 million hL (1st place), and wine consumption of 22.4 million hL, according to the International Organisation of Vine and Wine (OIV) Focus for 2018.^1^ Italy is also one of the richest countries in terms of the number of grape cultivars, because according to the Italian National Catalogue of Grapevine Varieties, over 500 cultivars currently compose the Italian ampelographic platform.^2^ Wine has had a direct and close relationship with Italian culture since the second century BC, and each region produces its own wine using local cultivars, also depending upon the characteristics of the territory, culinary habits, tradition, and human needs. The wine production of each region further evolved and developed unique characteristics over the centuries, to create the multi-oenological Italian culture of today, characterized by the presence of 525 origin wines, protected by intellectual property rights as either Denominazione di Origine Controllata e Garantita (DOCG; *n* = 74), Denominazione di Origine Controllata (DOC; *n* = 333), or Indicazione Geografica Tipica (IGT; *n* = 118).^3^

**Figure 1:**
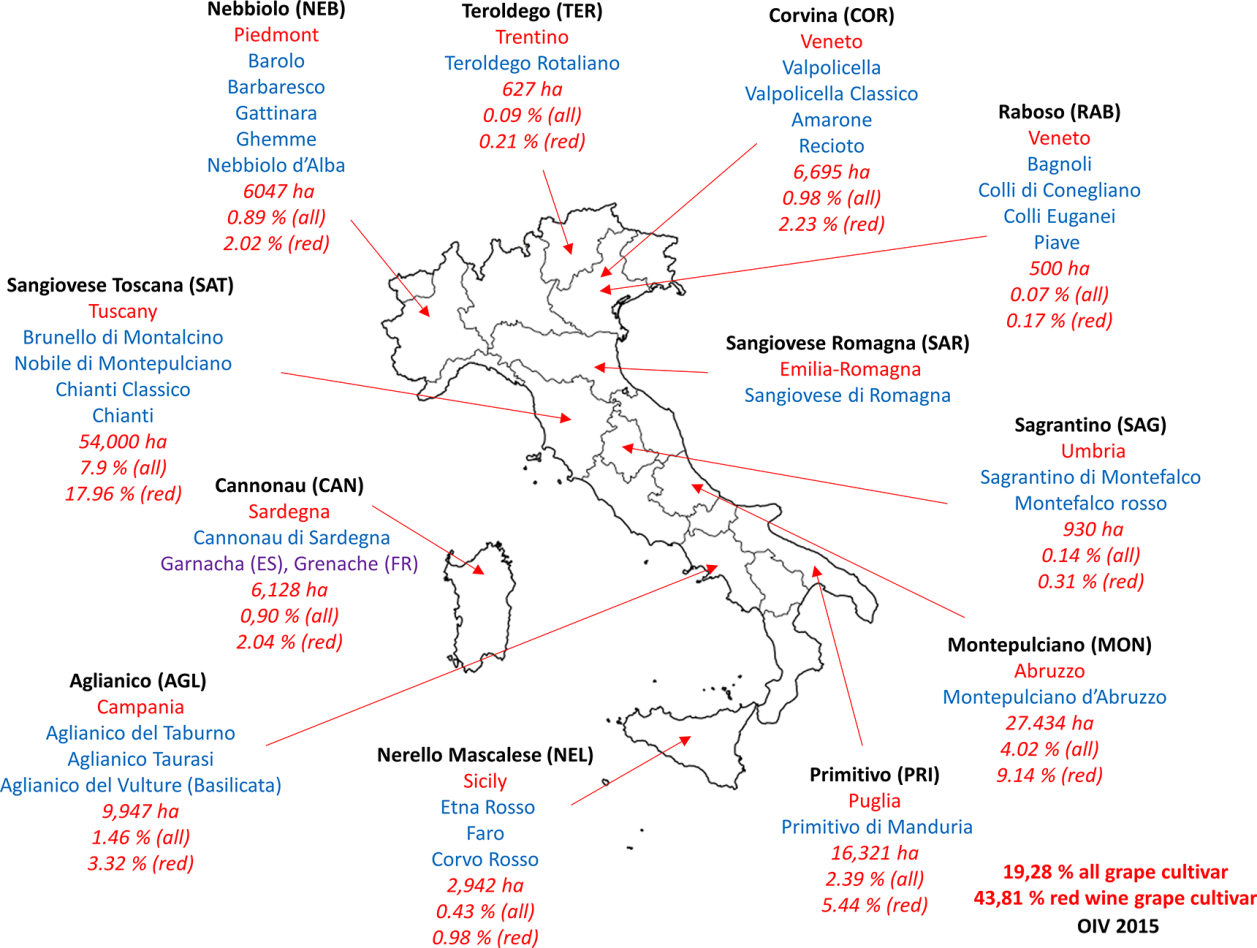
Distribution of the wine sample set according to their cultivar (black) and region (red). The principal denomination of origin of each cultivar/region is also shown (light blue). The cultivation area refers to the whole of Italy for each cultivar for the year 2015.^1^

In terms of the grapes used for wine production, Sangiovese is the major Italian cultivar, with 54 000 ha across the country (including Tuscany and Romagna), and is used to produce famous Italian wines, like Brunello di Montalcino and Chianti Classico. Nebbiolo is mainly cultivated in Piedmont, and iconic wines, like Barolo and Barbaresco, are produced from the harvest yielded by the 6047 cultivated ha. Corvina grapes (6695 ha) are used in the production of Amarone and Valpolicella in Veneto. In central and southern Italy, Montepulciano (27 434 ha) is the major red cultivar in Abruzzo, with Primitivo (16 321 ha) in Puglia, Aglianico (9947 ha) in Campania, and Cannonau (6128 ha) in Sardinia.^1^ Teroldego (627 ha), Raboso (∼500 ha), Sagrantino (930 ha), and Nerello Mascalese (2942 ha) are minor Italian cultivars, in terms of volume of production, and are cultivated mainly in limited areas of Trentino, Veneto, Umbria, and Sicily, respectively.^1^ In 2015, the above-mentioned cultivars accounted for 44% of the red grape vine-cultivated area of Italy and, therefore, constitute a representative portion of Italian oenological biodiversity (Figure 1).

Wine, being the final product of a long, multistep process, has one of the richest and most complex metabolomic fingerprints. Several targeted protocols based on analyzing polyphenols, volatiles, lipids, etc. have been applied to identify the differences between wines obtained from different grape cultivars as well as to understand the chemical and sensorial characters of monocultivar wines.^4−8^ In recent years, untargeted analytical approaches have proved to be a valid and appropriate alternative for the study of wine metabolome.^9−12^ Techniques such as liquid chromatography–mass spectrometry (LC–MS)-, gas chromatography–mass spectrometry (GC–MS)-, or direct injection Fourier transform ion cyclotron resonance–mass spectrometry (FTICR–MS)-based metabolomics have allowed for the identification of new wine metabolites,^13,14^ the discrimination of groups of wines,^14−17^ and the elucidation of the chemical reactions that occur during aging and storage,^13,14,16,18,19^ including in relation to packaging,^14^ thus providing novel insights into wine history^20^ and quality.^13,14,16,21,22^ Metabolomics developed and evolved as a consequence of the need to obtain a comprehensive characterization of the organic molecules in any biological system.^23^ Unlike the targeted methods, where the majority of the metabolites present in the matrix are ignored, in metabolomics, the aim is to achieve the widest possible metabolic coverage in an unsupervised manner, including unknown compounds. Consequently, the measured metabolites are by definition not pre-defined and method development and validation follows a different workflow from that of targeted analysis.

Some wines of the above-mentioned Italian cultivars have been subjected to untargeted LC–MS-based analysis, either alone or as groups together with 2–3 other cultivars, but the literature lacks studies combining a large part of the diversity of Italian red wines. Historically, the most promising markers for the chemical characterization of varietal wines were discovered by attempting to compare the presence of a few targeted metabolites in varietal wines. For example, a pioneering study^24^ based on the analysis of the variance of 20 organic acids and esters in six red wines led to the discovery that shikimic acid was associated with the cultivar and, more specifically, useful for distinguishing the Pinot Noir wines. It is expected that the application of an untargeted method, able to produce a semi-quantitative analysis of ca. 1000 metabolites, has the potential to support the discovery of several putative biomarkers of origin wines (pBOWs).

The primary objective of this project was to register, for the first time, the LC–MS metabolomic fingerprint of 11 monocultivar Italian red wines from 12 regions, representing a large portion of Italian red wine production and biodiversity. The secondary objective was to investigate the data set produced to obtain information regarding the metabolomic space similarity and dissimilarity between the studied wines and extract pBOWs. An additional objective was to make the data set publicly available to provide a resource for other researchers.

## Materials and Methods

### Wine Samples

A total of 110 Italian red wines, all of them monovarietal and vinified in 2016 using 11 different Italian grape varieties harvested in the corresponding main geographical areas of production (12 wine regions), were sampled directly from the producers. The wine sample set included 11 Teroldego (TER) from Trentino, 7 Corvina (COR) from Veneto, 10 Raboso Piave (RAB) from Veneto, 11 Nebbiolo (NEB) from Piedmont, 7 Sangiovese (SAT) from Tuscany, 12 Sangiovese (SAR) from Romagna, 10 Sagrantino (SAG) from Umbria, 9 Montepulciano (MON) from Abruzzo, 9 Cannonau (CAN) from Sardinia, 10 Aglianico (AGL) from Campania, 11 Primitivo (PRI) from Puglia, and 3 Nerello Mascalese (NER) from Sicily. The basic oenological information for the wines is provided in Table S1 and Figure S1 of the Supporting Information. The mid-infrared spectroscopy data can be found in the study by Parpinello et al.,^25^ and the sensorial analysis data can be found in the study by Piombino et al.^26^ Winemaking was carried out by each winery independently and according to their standard production practices. However, for each wine, the following specifications were followed: (a) the wines had to be obtained from a single grape variety; (b) the wines had to be fermented in stainless-steel vats; (c) fermentation had to be performed on an industrial scale; (d) sampling was to be performed before malolactic fermentation; (e) wines must not have any contact with oak; (f) 50 mg/L of free SO_2_ had to be added at the time of sampling, before bottling in dark glass bottles; and (g) Nomacorc Select Bio 500 (Nomacorc, France) closures had to be used. Sampling was performed in early 2017, and the wines were stored at 4 °C until analysis. All analyses were completed in a single batch, within 3 months of sampling.

### Ultra-Performance Liquid Chromatography–Quadrupole Time-of-Flight Mass Spectrometry (UPLC–QTOF MS) Analysis

Sample preparation was performed in accordance with a previously described protocol,^11^ and all of the steps up to LC–MS vial filling were performed in a nitrogen atmosphere. The wines were uncorked, and an aliquot was transferred to a 15 mL amber vial (filled to its capacity). Then, a pooled quality control (QC) sample was prepared by pooling 1 mL of each wine. Then, 1 mL of each wine sample/QC was diluted with 2 mL of Milli-Q sonicated water and was finally filtered with 0.2 μm polytetrafluoroethylene (PTFE) filters into a 2 mL amber vial (MS certificated) prior to LC–MS analysis. The samples were prepared and analyzed according to a randomized order (https://www.random.org/sequences/).

Analysis was performed in accordance with a previously described protocol.^11,13^ Waters Acquity UPLC coupled via an electrospray ionization (ESI) interface to Synapt HDMS QTOF MS (Waters, Manchester, U.K.) operating in W mode and controlled by MassLynx 4.1 was used. The column was a reversed phase (RP) ACQUITY UPLC, 1.8 μm, 2.1 × 150 mm, HSS T3 column (Waters); the column manager was set to 40 °C; the mobile phase flow rate was 0.28 mL/min; and the eluents were water (A) and methanol (B) both with 0.1% formic acid. The multistep linear gradient used was as follows: 0–1 min, 100% A isocratic; 1–3 min, 100–90% A; 3–18 min, 90–60% A; 18–21 min, 60–0% A; 21–25.5 min, 0% A isocratic; 25.5–25.6 min, 0–100% A; and 25.6–28 min, 100% A isocratic. The injection volume was 5 μL, and the samples were kept at 4 °C throughout the analysis. MS data were collected by separate runs in ESI positive and negative modes over a mass range of 50–2000 amu with a scan duration of 0.4 s, in centroid mode. The transfer collision energy and trap collision energy were set to 6 and 4 V, respectively. The source parameters were set as follows: capillary, 3 kV for positive scan and 2.5 kV for negative scan; sampling cone, 25 V; extraction cone, 3 V; source temperature, 150 °C; desolvation temperature, 500 °C; desolvation gas flow, 1000 L/h; and nebulizer gas, 50 L/h. External calibration of the instrument was performed at the beginning of each batch of analyses by direct infusion of a sodium formate solution (10% formic acid/0.1 M NaOH/acetonitrile at a ratio of 1:1:8), controlling the mass accuracy from *m*/*z* 40 to 2000 (less than 5 ppm) and mass resolution [over 14 000 full width at half maximum (fwhm)]. LockMass calibration was applied using leucine enkephalin solution (0.5 mg/L, *m*/*z* 556.2771 for positive and 554.2620 for negative ion mode) at 0.1 mL/min. The QC sample injections were used for the initial equilibration of the LC–MS system (4–5 injections) and controls at regular intervals (one QC sample injection every 6 real sample injections) during the sequence, in accordance with the QC flowchart.^11^ In total, the publicly available database (17.41 GB) includes 135 analyses (109 samples and 26 QC) for the ESI– mode and 134 analyses (110 samples and 24 QC) for the ESI+ mode (the system equilibration QC injections were excluded).

### Data Analysis

For quality control during the runs and data analysis, we used the principal component analysis (PCA) plots generated by Progenesis QI (version 2.4, nonlinear dynamics), by importing the raw files directly into the software, and checking the distribution/clustering of the QC injections.^11^ Progenesis QI parameters used for alignment were performed in default mode by Progenesis QI, with peak picking performed at the maximum level, and the first minute and the last 6 min of the run were excluded from data processing (only the 1–22 min range was used). Putative BOWs were considered the “compounds” that according to the Progenesis QI statistical analysis had a maximum fold range of ≥2 and analysis of variance (ANOVA) *p* value of ≤0.01. A maximum false discovery rate (FDR)-adjusted *p* value (or *q* value) threshold of 0.01 was applied for all putative biomarkers. Progenesis QI views as “compound” a group of isotopic and adduct features belonging to the same metabolite. The full lists of the pBOWs, including metadata, can be found in Tables S2 and S3 of the Supporting Information.

Annotation was performed manually by comparing retention times and mass spectra accuracy with a mass tolerance of 5 ppm based on the group’s previous experience with the specific instrumentation mass resolution^27^ and in accordance with the four levels described by Sumner et al.^28^ Of the 131 annotated metabolites (Table S4 of the Supporting Information), 78 were identified (level 1), 2 were putatively annotated (level 2), and 51 were putatively characterized (level 3).^28^ Putative annotations and characterizations were made using spectral features (mass difference less than 5 ppm of the theoretical value and the isotopic pattern) and literature information on chromatographic properties and mass spectra records from an external database, such as the Human Metabolome Database (HMDB, https://hmdb.ca/), and an internal wine metabolome database based on refs (13, 14, 27, and 29−33). Only a few annotated metabolites had a mass difference of 5 ppm greater that the theoretical value but less than 10 ppm, and this higher mass accuracy error was explained by the specific instrument capacities/characteristics (high or low *m*/*z* values and high or low peak intensity).^27^

Known wine metabolites previously annotated using the same protocol^11,13,14,29,30^ were integrated semi-manually using the TargetLynx tools of Waters MassLynx 4.1 software (Milford, MA, U.S.A.). The TargetLynx parameters were set at chromatogram mass window of 0.08 Da, retention time window of ±0.2 min, smoothing iterations of 1, and smoothing width of 2. Further statistical analysis was performed on these integrated peaks (Table S4 of the Supporting Information) using the MetaboAnalyst online platform, version 4.0 (http://www.metaboanalyst.ca/),^34^ without normalization, missing value estimation, and data transformation, using Pareto scaling. For the heatmap plots, the Euclidean distance and Ward clustering algorithm were used, applying the group average option.

Raw LC–MS data and other details are publicly available for download with accession number MTBLS1443 from the MetaboLights public repository (http://www.ebi.ac.uk/metabolights/).^21,35^

## Results and Discussion

The starting point of this study was to obtain a set of wine samples that was as representative as possible of the diversity of Italian red wine production in terms of both relevant varieties and areas of origin. As shown in Figure 1, the samples included regions of northern (Piedmont, Trentino, and Veneto), central (Tuscany, Emilia-Romagna, and Umbria), and southern (Campania, Puglia, and Abruzzo) Italy and its two largest islands (Sicily and Sardinia). In the case of Sangiovese, the most important red grape variety in Italy, two different production areas, namely, Tuscany and Emilia-Romagna, were considered. Wines were obtained from different wineries located in the production area, so that they could be considered true representations of not only the varietal characteristics but also the winemaking practices commonly adopted in each area at the winery level and in agreement with the rules of the specific denomination of origin. To avoid potential differences deriving from aging and storage practices, all samples were collected directly from the tank, without any previous contact with wood, and were bottled in the laboratory under the same conditions.

In recent years, the LC–MS protocol used has on various occasions proven its ability to register wine metabolome and generated new hypotheses.^11,13,14,30^ As stated by this protocol, one of the most crucial issues in untargeted LC–MS analysis is to inject all samples in a single batch. As a result of this methodological constraint, in this study, it was decided to analyze only the wines produced in one harvest. The number of biological replicates, i.e., different wines produced from different vineyards and/or different wineries, was in the range of 7–12 (mean of 9.7) for all of the wine regions, with the sole exception of Nerello Mascalese from Sicily, for which only three wine samples were obtained.

**Figure 2:**
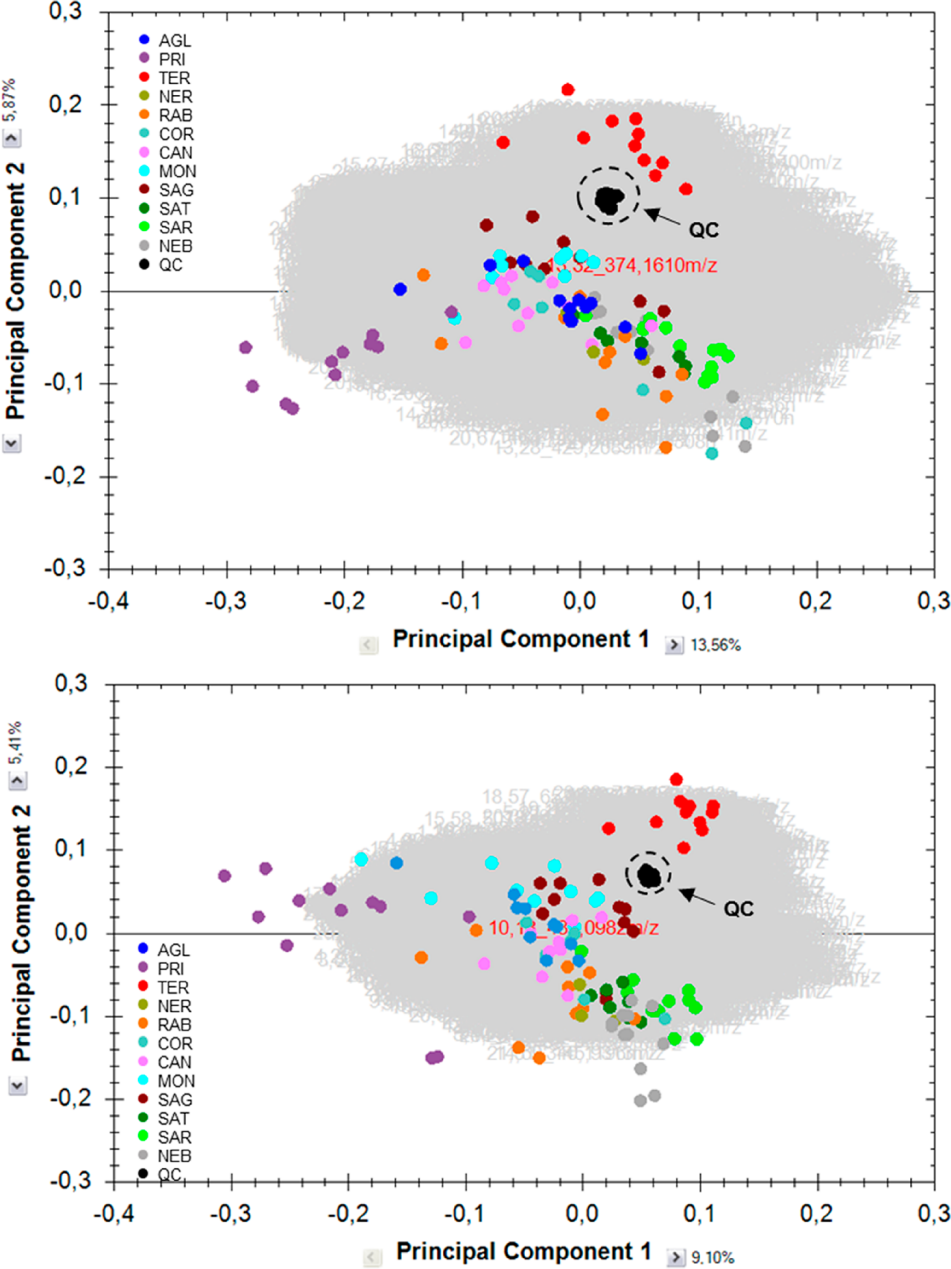
PCA plots of all of the wines in (top) ESI+ and (bottom) ESI–. AGL, Aglianico; PRI, Primitivo; TER, Teroldego; NER, Nerello Mascalese; RAB, Raboso; COR, Corvina; CAN, Cannonau; MON, Montepulciano; SAG, Sagrantino; SAT, Sangiovese Tuscany; SAR, Sangiovese Romagna; NEB, Nebbiolo; and QC, quality control.

In accordance with the workflow adopted in our laboratory, before any further data analysis, it is important to verify the quality of the data set. Figure 2 shows the PCA plots of the distribution of sample injections according to multivariate and unsupervised PCA. The PCA plot of the ESI+ analysis was performed using 11 274 features, with the ESI– analysis using 7397 features, and in both cases, the QC sample injections, injected throughout the sequence, formed a tight cluster, proving the reliability of the measure, in terms of the absence of fluctuations for samples injected at different time points. According to this unsupervised analysis, it was possible to notice that Teroldego and Primitivo wine groups had a metabolomic fingerprint that was very different from the other wines.

**Figure 3:**
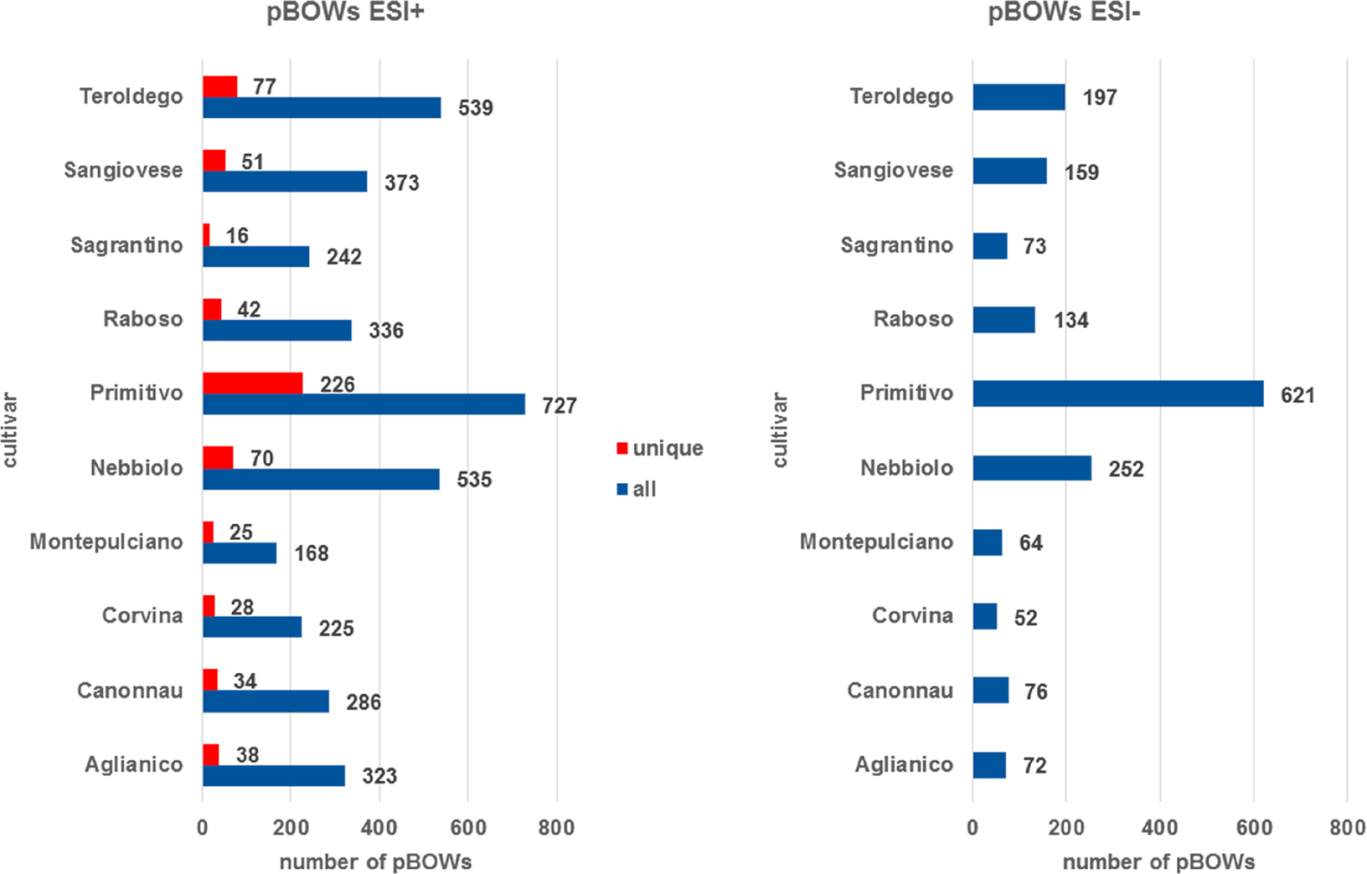
Number of pBOW features for each cultivar in ESI+ and ESI–. The pBOWs that help to discriminate the cultivar from all of the others are unique.

**Figure 4:**
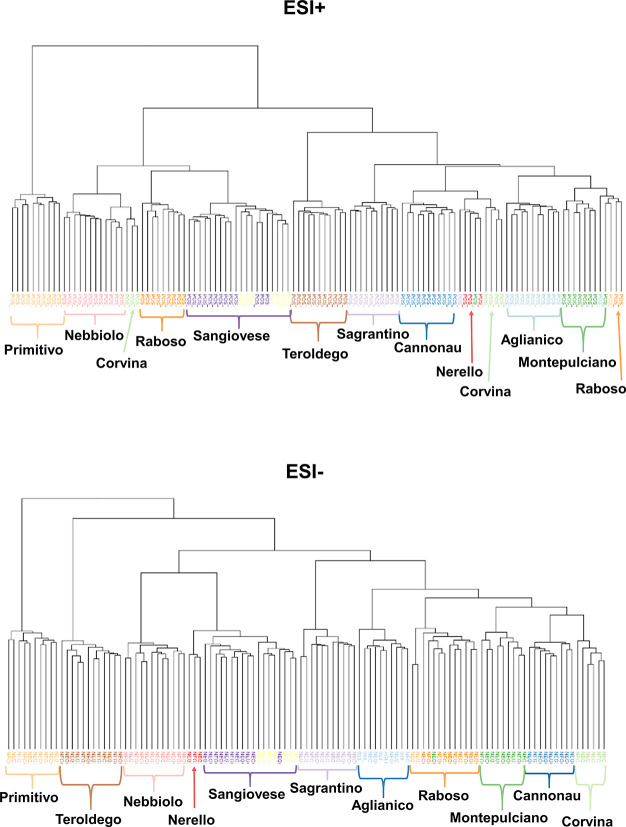
Clustering of the wines according to the markers in ESI+ and ESI–.

To investigate the metabolites that differentiated each wine group from the others, we used supervised data analysis tools. Using the Progenesis QI ANOVA tool, the metabolomic fingerprint of each wine group was compared to all of the other groups; therefore, a subgroup of features was created using only the features with a *p* value of ≤0.01 and fold change of ≥2. The different lists were merged and created Tables S2 and S3 of the Supporting Information. The ESI– analysis included 621 pBOWs, and the ESI+ analysis included 1735 pBOWs. Figure 3 shows the main outcome of this data analysis. For the ESI+ analysis, it was also possible to detect pBOWs that were unique to each group of wines, whereas this was not possible for the ESI– analysis, because Primitivo included all of the pBOWs and did not have any unique pBOWs. Indeed, both ESI– and ESI+ analyses showed that Primitivo had the highest number of pBOWs. This result was also in accordance with both the PCA plots (Figure 2), where Primitivo samples are separated from the other cultivars by PC1, and the hierarchical cluster analysis (Figure 4), where Primitivo samples are the first group of samples to break away from the others. More specifically, Primitivo has 727 feature pBOWs (226 of them unique) for ESI+ and 621 for ESI–. Teroldego and Nebbiolo also had a large number of pBOWs, whereas Montepulciano and Corvina had the smallest number of pBOWs.

The hierarchical cluster analysis (Figure 4) showed that the Primitivo group was the one that differed most for both the ESI– and ESI+ analyses. A second cluster in ESI+ included Nebbiolo, Corvina, Raboso, and Sangiovese wines. This behavior should be attributed to the fact that these cultivars are known for their light red color^5^ and because, in ESI+ mode, the positively charged anthocyanins give a very intense signal. Therefore, the clustering observed here was most likely strongly driven by the red-colored, positively charged anthocyanins. The finding that Teroldego, a cultivar very rich in anthocyanins,^5^ formed a cluster alone supports this hypothesis. These findings suggested that we should investigate anthocyanins and related pigments in further detail. In the ESI– analysis, Teroldego was the second most distant cluster, whereas Nebbiolo, Nerello, and Sangiovese once again clustered as nearest neighbors in the dendrogram (Figure 4).

The pBOW annotation process showed that several of the metabolites belong to the chemical classes of polyphenols, amino acids, dipeptides, tripeptides, bound terpenoids, sugars, and organic acids (Tables S2 and S3 of the Supporting Information). It was therefore decided to take advantage of the annotation achieved previously using the same protocol in oenological studies and to study these groups of known metabolites in greater depth.^11,13,14,21,27,30^ With this aim, we returned to the raw files and integrated a large number of metabolites. This integration process was independent of the Progenesis QI workflow and, therefore, provided a way to manually check the possible presence of false positive and false negative markers. The integrated area peak table was then uploaded to the MetaboAnalyst platform for further statistical analysis and data visualization.

**Figure 5:**
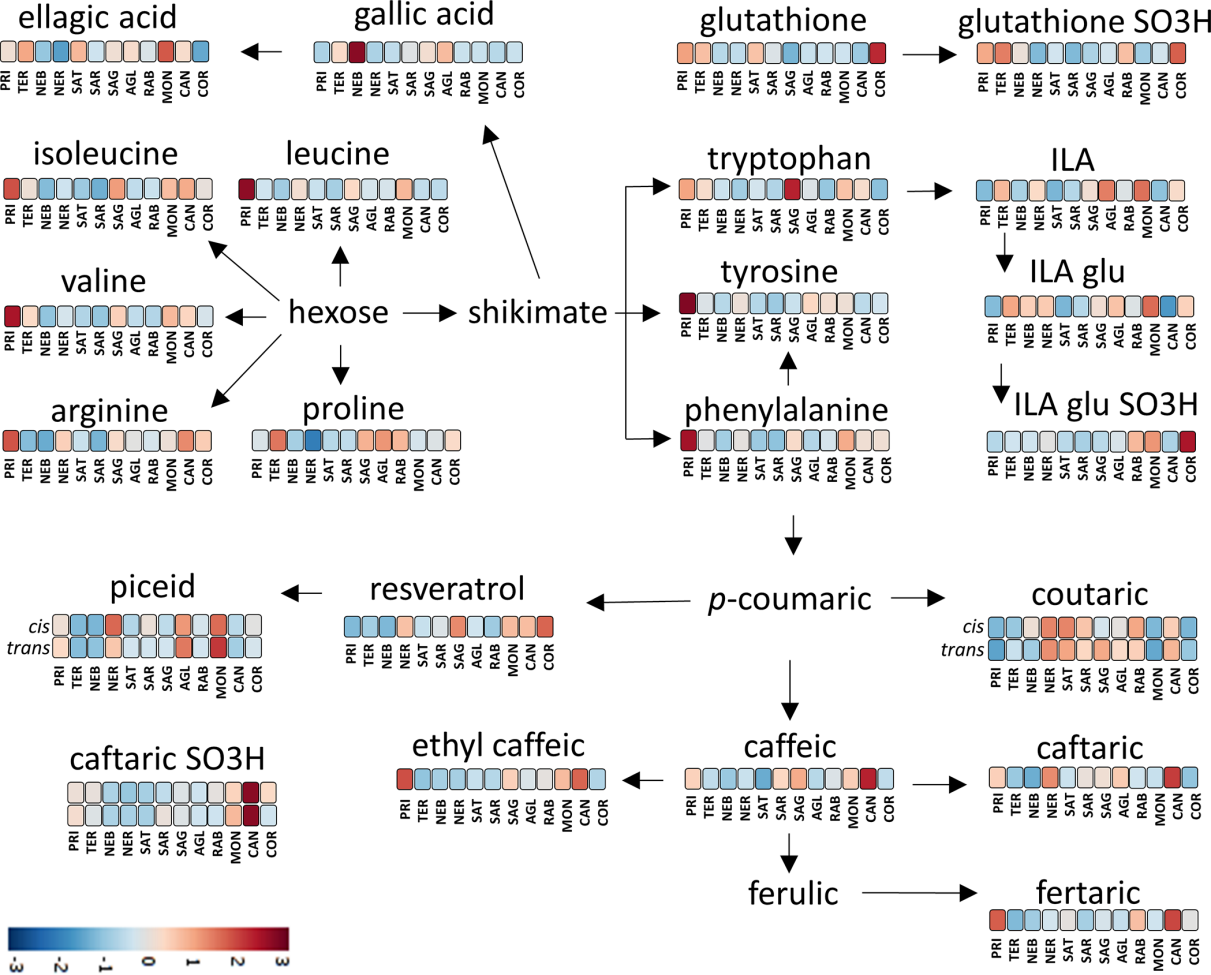
Biosynthesis and synthesis of N-containing metabolites, hydroxycinnamates, and stilbenoids annotated in this study. The colors refer to the heatmap of Figure S1 of the Supporting Information and provide a comparison of the concentration of each metabolite between the various monocultivar wine groups. The heatmap was constructed using Pareto scaling and Euclidean distance. AGL, Aglianico; PRI, Primitivo; TER, Teroldego; NER, Nerello Mascalese; RAB, Raboso; COR, Corvina; CAN, Cannonau; MON, Montepulciano; SAG, Sagrantino; SAT, Sangiovese Tuscany; SAR, Sangiovese Romagna; and NEB, Nebbiolo.

**Figure 6:**
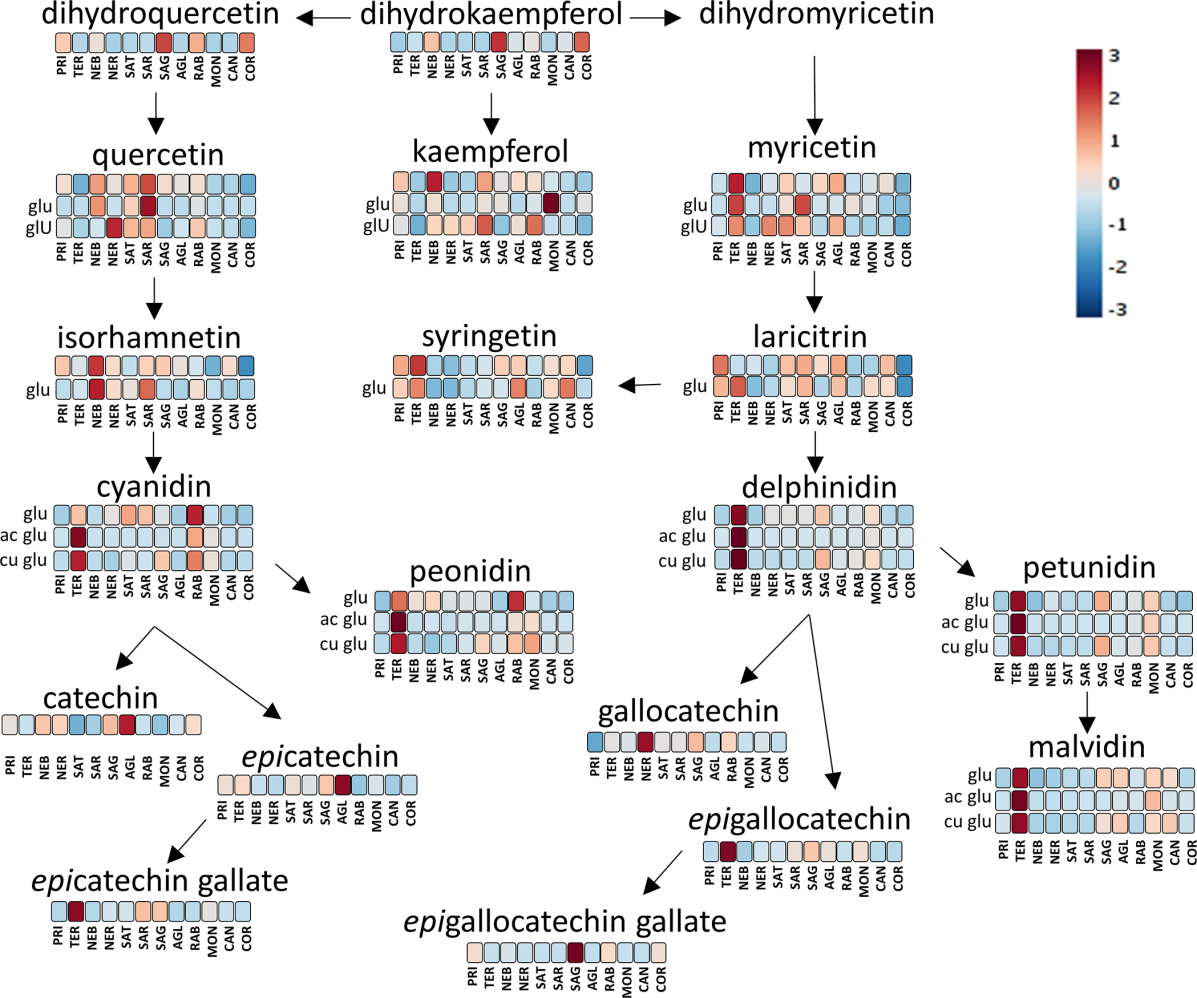
General pattern for flavonoid biosynthesis, with the metabolites annotated in this study. The colors refer to the heatmap of Figure S1 of the Supporting Information and provide a comparison of the concentration of each metabolite between the various monocultivar wine groups. The heatmap was constructed using Pareto scaling and Euclidean distance. AGL, Aglianico; PRI, Primitivo; TER, Teroldego; NER, Nerello Mascalese; RAB, Raboso; COR, Corvina; CAN, Cannonau; MON, Montepulciano; SAG, Sagrantino; SAT, Sangiovese Tuscany; SAR, Sangiovese Romagna; and NEB, Nebbiolo.

**Figure 7:**
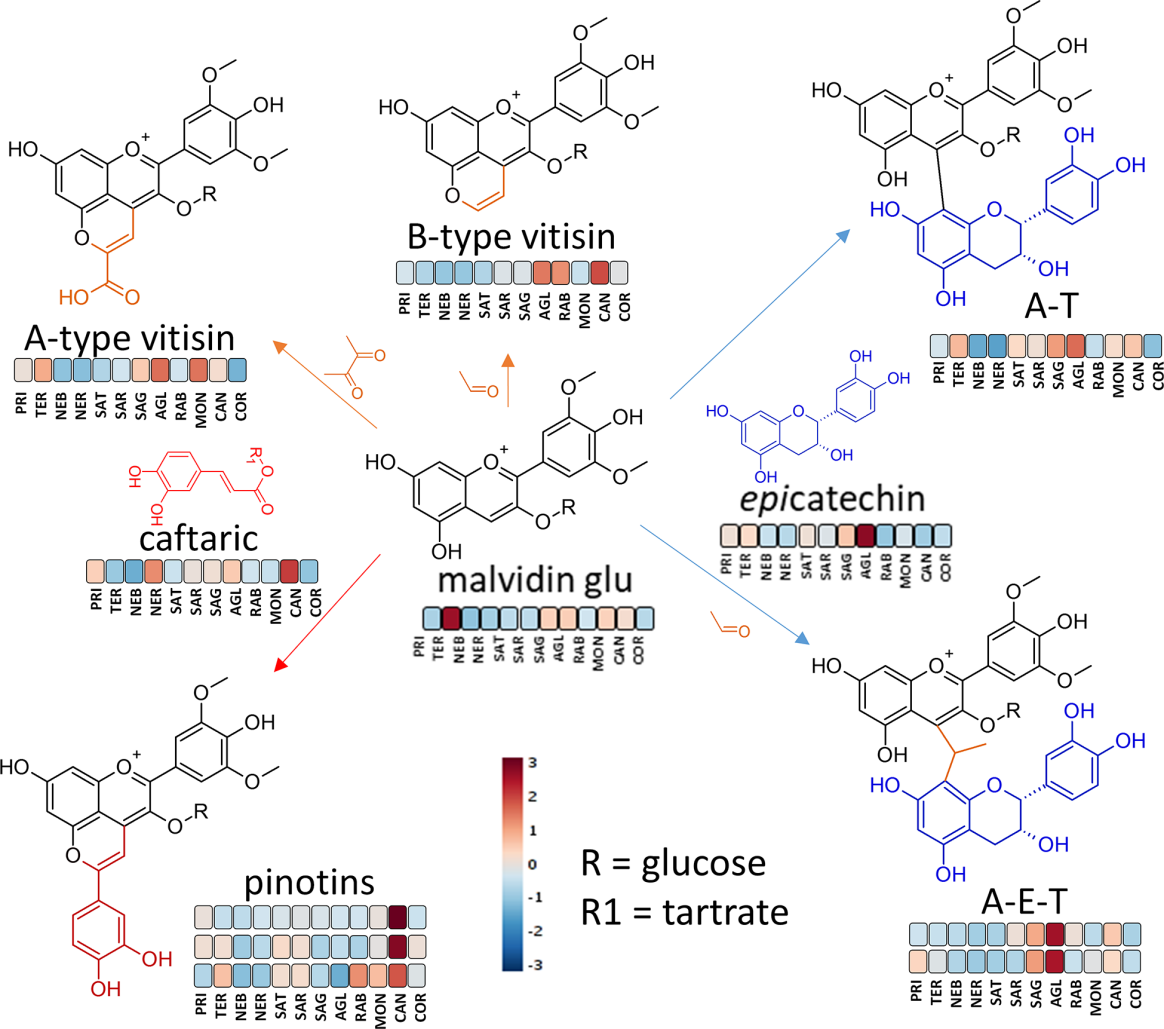
Generic diagram with the major reaction in which anthocyanins participate in wine. The colors refer to the heatmap of Figure S1 of the Supporting Information and provide a comparison of the concentration of each metabolite between the various monocultivar wine groups. The heatmap was constructed using Pareto scaling and Euclidean distance. AGL, Aglianico; PRI, Primitivo; TER, Teroldego; NER, Nerello Mascalese; RAB, Raboso; COR, Corvina; CAN, Cannonau; MON, Montepulciano; SAG, Sagrantino; SAT, Sangiovese Tuscany; SAR, Sangiovese Romagna; and NEB, Nebbiolo.

Figures 5–7 show the (bio)synthetic pathway of several metabolites of oenological interest that were annotated and detected as markers in this study. For each metabolite, data from the heatmap of Figure S1 of the Supporting Information is also shown, to compare the relative concentration of each metabolite in the different wine groups. With regard to the amino acids included in Figure 5, Primitivo was the group with the highest amount of leucine, arginine, tyrosine, valine, and phenylalanine. At the opposite end of the spectrum, the wine groups with the smallest amounts of the same amino acids were Nebbiolo and Sangiovese. It should be taken into consideration that, during alcoholic fermentation, yeasts may consume most of the amino acids as a nitrogen source.^36^ If this is so, common oenological practices, such as the addition of inorganic and/or organic nitrogen to support yeast growth, would strongly affect the concentration of amino acids in wine.^36^ Because the wines from each group originated from different wineries following different winemaking practices, it cannot be ruled out that amino acids could act as markers to discriminate between wines obtained from different cultivars. In the past, the amino acid profile has been proposed as a tool for wine discrimination.^36−38^ Proline is the only amino acid not consumed by yeast in anaerobic conditions,^36^ a characteristic that makes it suitable for use in food fraud analysis.^39^ According to our results, Primitivo wines showed a relatively low concentration for this amino acid, with Teroldego showing the highest and Nerello showing the lowest.

Moreover, several di- and tripeptides were tentatively annotated (third level annotation) as markers. According to the nitrogen rule/principle in MS, odd *m*/*z* values indicate organic compounds with an odd number of nitrogen atoms (at least one) and even *m*/*z* values indicate organic compounds with zero or an even number of nitrogen atoms. Of course, this rule is valid for organic compounds containing exclusively H, C, N, O, Si, P, S, and halogen, and for high-resolution mass spectrometers, it is more accurate for *m*/*z* values below 500. Primitivo wine pBOWs included several ions with odd *m*/*z* values (Tables S2 and S3 of the Supporting Information) and had the highest concentrations in several amino acids (Figure 5), and the tentatively annotated compounds included di- and tripeptides. If these findings are characteristic for Primitivo, further experiments are necessary to validate this hypothesis and better understand the composition of Primitivo wines and the contribution of the cultivar and its terroir in determining this unusual richness in nitrogen compounds. Lately, Sherman et al.^40^ discovered that the sensorial quality of wine has a positive correlation with markers annotated as di- and tripeptides. To validate the hypothesis that the amino acid profile could be used to distinguish the cultivar in wines, the analyses will have to be conducted on wines produced in more than one harvest as well as the use of wines produced under the same winemaking conditions and under well-controlled agronomical conditions. Indeed, it is well-known that, in addition to the cultivar, the terroir (fertilization with nitrogen, grape maturity, climate, and sanitary status) can also greatly influence the concentration in nitrogen-containing compounds.^41^

Primitivo and Sagrantino were the wines with the highest tryptophan content, whereas Sangiovese, Raboso, and Nebbiolo had the lowest tryptophan content. Conversely, Sangiovese wines were the richest in tryptophol, the Ehrlich reaction tryptophan product formed during alcoholic formation, and Primitivo wines were the poorest in tryptophol (Figure 5). This was an indication that tryptophan was used by the yeast during the alcoholic fermentation of Sangiovese wines.^36^ The lower presence of tryptophol in Primitivo wines was expected, because the Ehrlich pathway is not a preferred way of nitrogen assimilation in the presence of an abundant amino acid content in the juice. Moreover, we found that Sangiovese wines were also the richest in sulfonated tryptophol (Figure S1 of the Supporting Information), which is a product of the sulfonation of tryptophol, and its formation is favored by oxygen and lower pH.^14,32,42^ Primitivo wines were also the richest in two other N-containing metabolites, tryptophan products produced during the alcoholic fermentation: *N*-acetyl-tryptophan ethyl ester and tryptophan ethyl ester.^42^ During the Primitivo winemaking process, tryptophan would appear to transform into these two ethyl esters and not to the fusel alcohol (tryptophol). In line with our previous experience,^32^ the same also applies for tyrosine (Figure S1 of the Supporting Information).^32,42^

In grapes, tryptophan is transformed into indole lactic acid (ILA) and its glucosides (ILA-glu), and later these two metabolites can react with SO_2_ in the wine and yield the corresponding sulfonated products (ILA-SO_3_H and ILA-glu-SO_3_H).^14,32^ The concentrations of ILA and ILA-glu depend upon the cultivar and climate, and in our experiment, Montepulciano, Aglianico, and Teroldego showed the highest concentrations (Figure 5). The Corvina wines had the highest concentration of sulfonated ILA-glu-SO_3_H, followed by Montepulciano and Raboso. The formation of sulfonated indoles in wine is strongly linked with oxygen.^14,32^

Glutathione is a tripeptide present in grapes that can also be added to wine (mainly white wines) as an antioxidant to preserve the aromatic compounds.^36^ It was recently proven that, in the presence of SO_2_, glutathione can produce its sulfonated analogue. The presence of oxygen can also favor this reaction.^14^ Corvina was the group of wines with the highest concentration of both glutathione and its sulfonated analogue (Figure 5).

Through the phenylpropanoid pathway, grapevine is able to synthesize several polyphenols pertaining to different families. One of the main families is the hydroxycinnamates, which include coutaric, caftaric, and fertaric acids. Sangiovese, Nerello, Raboso, and Cannonau were the wines with the highest concentration in monosubstituted (i.e., one −OH to the aromatic ring) coutaric acid, whereas disubstituted caftaric acid, sulfonated caftaric acid, caffeic acid, and fertaric acid are typical of Cannonau (Figure 5). This is likely a characteristic derived from the cultivar, because Cannonau grapes belong to the Grenache/Garnacha grape family, known to have one of the highest hydroxycinnamate content among *Vitis vinifera* grape cultivars.^43^

Primitivo showed the lowest concentrations of coutaric acid, medium concentrations of caftaric acid, and highest concentrations of fertaric acid. This could be a characteristic that genetically distinguishes the pathway that produces hydroxycinnamates in Primitivo from the other cultivars analyzed in this study. As for stilbenoids, of which the concentration depends upon the cultivar and possible plant stress, such as fungal infection,^44^ Montepulciano showed the highest concentrations for the glucosidic forms.

Figure 6 summarizes another important branch of the general pathway for the synthesis of polyphenols, where the flavonoids are classified according to the number of B-ring substitutes. This figure includes the families of flavanonols (dihydroquercetin, dihyrokaempferol, and dihydromirycetin), flavonols (quercetin, isorhamnetin, kaempferol, syringetin, myricetin, and laricitrin), anthocyanins (cyanidin, peonidin, delphinidin, malvidin, and petunidin), and flavanols (catechin, epicatechin, gallocatechin, etc.). The kaempferol pathway has just one substitute, with quercetin having two substitutes and myricetin having three substitutes. It is known that the ratio between these three chemical groups is genetically controlled and often used to distinguish cultivars.^4,5^ Teroldego was characterized by the highest concentration in the trisubstitute families, in other words the derivatives of myricetin, delphinidin, petunidin, and malvidin. Teroldego wines also appeared to be those with the highest content of all anthocyanins. Sangiovese wines were the richest in quercetin, followed by Nebbiolo and Nerello. These data are in agreement with a previous study on grapes, where all grape vines were cultivated in the same vineyard and under the same conditions.^5^ According to Mattivi et al.,^5^ myricetin had the highest percentage between all flavonols for Teroldego (74%) and Sagrantino (82%), whereas quercetin had the highest percentage for Sangiovese (67%) and Nebbiolo (70%). The same study, which included all of the cultivars considered in this study with the exception of Nerello Mascalese, is in agreement with our findings regarding the rich anthocyanin content of Teroldego. In recent years, Sangiovese wines have suffered from a problem of instability regarding quercetin (and other flavonols), generating floating flakes in the bottled wine.^45^ The chemical analysis demonstrated that the major component of these flakes is quercetin aglycon, which suggests that this occurs in wines with a high quercetin content.^45^ To the best of our knowledge, this problem has never been previously reported in Nebbiolo or Nerello wines, which according to our results had the highest concentration of quercetin after Sangiovese.

Nebbiolo was also the group of wines with the highest content of isorhamnetin, which is the methylation product of quercetin and is disubstituted in the B ring. This finding was also in agreement with Mattivi et al.,^5^ where isorhamnetin represented 15% of all flavonols for Nebbiolo. After Teroldego, Raboso was the second group of wines in terms of cyanidin and peonidin contents. For the trisubstitute anthocyanins, after Teroldego, Montepulciano and Sagrantino were the richest cultivars, followed by Aglianico and Cannonau.

With regard to monomeric flavanols, Aglianico was the richest group for catechin and epicatechin, followed by Sagrantino and Teroldego for epicatechin and Sagrantino, Nerello, Nebbiolo, and Corvina for catechin. Teroldego was the richest group for epicatechin gallate, followed by Sagrantino and Sangiovese from Romagna. Nerello was the richest in gallocatechin, and Teroldego was the richest in epigallocatechin. Finally, Sagrantino was also the richest for epigallocatechin gallate (Figure 6). Flavanols are an important family of polyphenols in wine because, among other things, they influence the astringency and bitterness of the wine. According to Cheynier et al.,^46^ epicatechin is more bitter than catechin and galloylation increases astringency.

Wine is not just a grape product but involves a complex technological process (alcoholic fermentation, malolactic fermentation, etc.), and each step enriches and modifies the metabolomic fingerprint of the wine. Additionally, wine metabolites evolve continuously during aging. Anthocyanins, which are the metabolites responsible for the red color of the wines (and many other foods and flowers), participate in a number of reactions during wine aging, leading to the production of several classes of wine pigments. As Figure 7 shows, Teroldego was the group with the highest grape anthocyanin content, but Aglianico was richest in direct-linked and ethyl-bridged linked flavanols–anthocyanins, probably because of its higher epicatechin content. Sagrantino, Cannonau, and Primitivo were also particularly rich in ethyl-bridged flavanols–anthocyanins. After Aglianico, the richest groups in directed-linked flavanols–anthocyanins were Sagrantino, Teroldego, Cannonau, and Sangiovese. Cannonau, which was the richest in caftaric acid (Figure 5), was also the richest group for certain pinotins, the products of the condensation reaction between hydroxycinnamates and anthocyanins (Figure 7). Finally, the product of the reaction between malvidin 3-glucoside and acetaldehyde, B-type vitisin, was to found to be more characteristic of Cannonau, Raboso, and Aglianico, whereas the product of the reaction between malvidin 3-glucoside and pyruvic acid characterized the Montepulciano, Aglianico, Sagrantino, and Teroldego groups (Figure 7).

**Figure 8:**
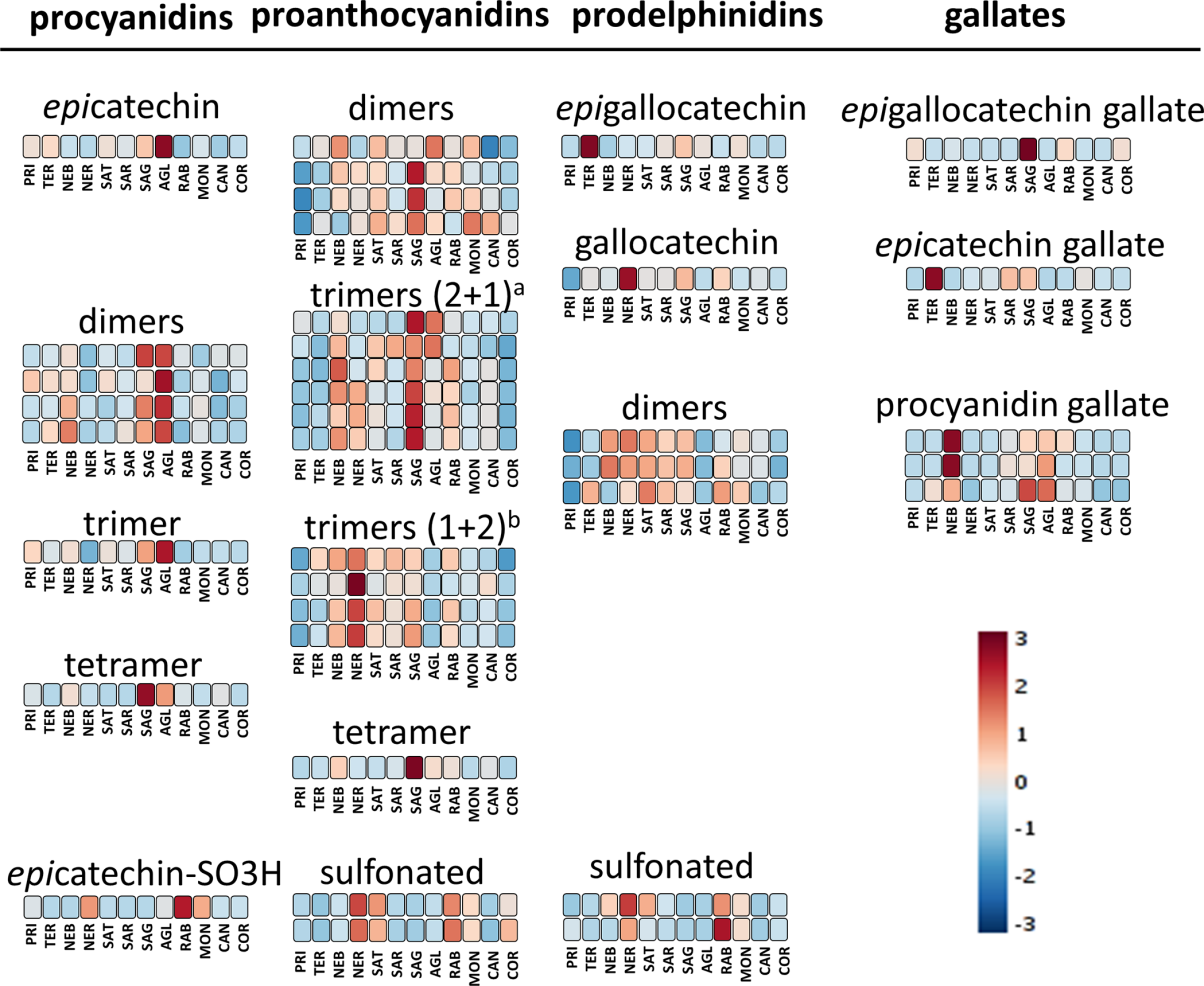
Variation of the annotated monomeric and oligomeric flavanols according to the various monocultivar wine groups. The classification is based on B-ring substitution. The colors refer to the heatmap of Figure S1 of the Supporting Information and provide a comparison of the average concentration of each metabolite within each of the various monocultivar wine groups. The heatmap was constructed using Pareto scaling and Euclidean distance. AGL, Aglianico; PRI, Primitivo; TER, Teroldego; NER, Nerello Mascalese; RAB, Raboso; COR, Corvina; CAN, Cannonau; MON, Montepulciano; SAG, Sagrantino; SAT, Sangiovese Tuscany; SAR, Sangiovese Romagna; and NEB, Nebbiolo. (a) Two disubstituted and one trisubstituted block. (b) One disubstituted and two trisubstituted block.

One key objective of this project was to study the tannins of the Italian red wines obtained from the grapes; therefore, all of the wines were prepared without any tannin addition or contact with wooden barrels. Figure 8 provides a comparison of the wine groups for different monomeric, dimeric, trimeric, and tetrameric flavanols and also includes some monomeric sulfonated flavanols. Moreover, the metabolites were divided into four families based on their B-ring substitutions: (a) procyanidins, only constituted by the disubstituted catechin and epicatechin; (b) proanthocyanidins, which have at least one trisubstituted gallocatechin or epigallocatechin and one disubstituted catechin or epicatechin; (c) prodelphinidins, constituted solely by trisubstituted gallocatechin and epigallocatechin; and (d) gallates, which include at least one galloyl moiety. According to previous studies, the polymerization of tannins decreases the bitterness and dimers, trimers, and tetramers are perceived as being more bitter than astringent. As polymerization increases, astringency initially increases (oligomeric tannins), but as polymerization further increases, astringency decreases (polymeric tannins).^46^

The Aglianico group was the richest in procyanidin-type tannins, followed by Sagrantino and Nebbiolo. These three cultivars are known to produce wines with an astringent character. Conversely, Cannonau, Corvina, Montepulciano, Raboso, and Nerello showed the lowest procyanidin contents. Sagrantino wines were also the richest in mixed proanthocyanidins, followed by Nerello and Nebbiolo, whereas Primitivo, Corvina, and Teroldego had the lowest content. With regard to prodelphinidins, Sagrantino, Sangiovese, Nerello, Nebbiolo, and Teroldego were the richest and Primitivo, Corvina, and Cannonau were the poorest. Sagrantino, Aglianico, Teroldego, and Nebbiolo were the richest in galloylated flavanols, while Primitivo, Corvina, Cannonau, and Nerello contained the lowest amounts. Raboso, Nerello, Sangiovese from Tuscany, and Montepulciano were the wines with the highest concentration of sulfonated tannins (Figure 8).

Generally, this analytical survey on the untargeted metabolomic fingerprint of 11 Italian single-cultivar red wines, considered together for the first time, highlighted the huge diversity in the composition of these Italian origin wines and generated hypotheses that will need to be validated in the future with targeted approaches. Primitivo was the wine group with the most distinctive metabolome, being characterized by the highest content in several amino acids (tyrosine, phenylalanine, arginine, valine, leucine, and isoleucine), and the lowest proline content. In agreement with these findings, Primitivo wines were also characterized by a large number of N-containing metabolites. One additional characteristic of Primitivo was the increased level of methylation of both hydroxycinnamates and flavonols. Finally, Primitivo wines had a low anthocyanin and oligomeric flavanol content.

Teroldego was another wine group with a distinctive metabolomic fingerprint, characterized by the highest content of anthocyanins, in particular anthocyanins with three B-ring substitutions. Increased B-ring substitution in Teroldego was also observed for flavonols.

Nebbiolo wines were poor in amino acids, hydroxycinnamates, anthocyanins, and their derivatives but rich in kaempferol, isorhametin, and quercetin (the second richest group in quercetin after Sangiovese). Condensed tannins were detected in high concentrations in the Nebbiolo wines, as were procyanidin gallates and gallic acid. This high galloylation could perhaps explain the astringent character of renowned Nebbiolo wines as Barolo and Barbaresco.^47,48^

Aglianico wines were the richest in catechin, epicatechin, procyanidins, A-type vitisin, B-type vitisin, and the products of reactions between anthocyanins and flavanols (both ethyl- and direct-linked). Aglianico samples did not exhibit particularly high levels of anthocyanins, possibly as a result of the high rate of reaction with flavanols, resulting in the synthesis of stable anthocyanin adducts and, therefore, a more stable color. The high monomeric and oligomeric procyanidin contents could also be responsible for the highly astringent character of Aglianico wines.^49,50^

Sangiovese, the most widespread Italian cultivar, was similar to Nebbiolo and Nerello on the ESI– analysis, whereas for ESI+, it showed a metabolite profile similar to that of Nebbiolo and Raboso. If we consider all of the wine groups, Sangiovese wines were characterized by the B-ring disubstituted flavonols (quercetin derivatives) and anthocyanins (cyanidin 3-glucoside) and the disubstituted hydroxycinnamates (coutaric acid). The tannins of Sangiovese were rich in proanthocyanidins/prodelphinidins with trisubstituted flavanols (gallocatechin and/or epigallocatechin units), whereas the Sangiovese wines from Tuscany were rich in sulfonated oligomeric flavanols. Finally, Sangiovese wines were poor in amino acids and N-containing metabolites. Overall, Sangiovese wines from Tuscany and Romagna were close and had a very similar metabolome.

Cannonau wines were characterized by various caffeic acid metabolites (caftaric acid, caffeoyl derivatives, sulfonated caftaric acid, and pinotins). They were also rich in B-type vitisin, arginine, and B-ring methylated flavonoids (syringetin, laricitrin, and malvidin derivatives) but relatively poor in tannins.

Sagrantino wines showed the highest content of tryptophan and had intermediate contents for the other amino acids. Oligomeric tannins were generally high in Sagrantino, both direct- and ethyl-linked flavanols–anthocyanins, and had the highest levels of proanthocyanidins and epigallocatechin gallate. Sagrantino wines were also characterized by the highest flavanonol (dihydroxykaempferol and dihydroxyquercetin) content and by relatively higher levels of coutaric acid than caftaric and fertaric acids.

Corvina wines were the least homogeneous group, with generally low polyphenol levels (except flavanonols) and the highest sulfonated glutathione and sulfonated indole lactic acid glucoside contents. Raboso wines were characterized by disubstituted anthocyanins, cyanidin 3-glucoside and peonidin 3-glucoside, and sulfonated tannins. The Montepulciano group was characterized by acetylated anthocyanins, indole lactic acid and its glucoside, and ellagic acid.

To conclude, the use of a robust untargeted LC–MS-based analytical protocol together with a targeted sampling protocol covering a large portion of Italian oenological biodiversity produced an interesting publicly available database. Of the 11 monocultivar, single-vintage red wines investigated, Primitivo, Teroldego, and Nebbiolo had the highest number of pBOWs, and a second group comprised Sangiovese, Aglianico, Cannonau, and Raboso. Primitivo and Teroldego had the most distinctive metabolomic fingerprint, while Sangiovese and Nebbiolo had very similar metabolomes, as did Montepulciano and Cannonau. Of the pBOWs, we annotated several N-containing metabolites (amino acids, di- and tripeptides, etc.), showing that these metabolites could be instrumental to understanding and exploiting wine diversity. Primitivo wines, in particular, were very rich in N-containing metabolite tentative markers. The wines with the metabolome richest in condensed tannins were Sagrantino, Nebbiolo, and Aglianico. Teroldego was characterized by the highest anthocyanin content, followed by Raboso, Montepulciano, Sagrantino, and Aglianico. Sangiovese, Nebbiolo, Nerello, and Raboso were characterized by flavonoids with a disubstituted B ring, and Primitivo, Teroldego, Aglianico, Cannonau, and Montepulciano were characterized by flavonoids with a trisubstituted B ring. In parallel, monosubstituted hydroxycinnamates characterized Sangiovese, Nerello, and Raboso, and di- and trisubstituted hydroxycinnamates characterized Primitivo and Cannonau wines. As expected, the polyphenol pathway offers many tools for understanding the metabolomic diversity of the wines. Moreover, even if all wines had the same total SO_2_, this wine preservative reacts in a different manner with the metabolites of each wine. In Corvina, Montepulciano, and Raboso, it reacts with ILA-glu; in Teroldego, Corvina, Raboso, and Primitivo, it reacts with glutathione; and in Nerello, Sangiovese, and Raboso, it reacts with flavanols. Both raw and analyzed data are publicly available, to help other researchers to better understand Italian oenological diversity and quality.

## Supporting Information Available

Wine meta-information and basic oenological analysis (Table S1) (XLSX)
Putative marker list for the ESI– analysis, including information regarding the annotation, annotation level, statistical data, and the group(s) of wines that were markers (Table S2), and putative markers list for the ESI+ analysis, including information regarding the annotation, annotation level, statistical data, and the group(s) of wines that were markers (Table S3) (XLSX)
Annotated metabolite metadata and semi-quantification results (Table S4) (XLSX)
Heatmap of all annotated metabolites used for Figures 3–8 (Figure S1) (PDF)

## Abbreviations Used

AGL: Aglianico
PRI: Primitivo
TER: Teroldego
NER: Nerello Mascalese
RAB: Raboso
COR: Corvina
CAN: Cannonau
MON: Montepulciano
SAG: Sagrantino
SAT: Sangiovese Tuscany
SAR: Sangiovese Romagna
NEB: Nebbiolo
QC: quality control
pBOWs: putative biomarkers of origin wines
LC: liquid chromatography
MS: mass spectrometry
FTICR: Fourier transform ion cyclotron resonance
UPLC–QTOF MS: ultra-performance liquid chromatography–quadrupole time-of-flight mass spectrometry
PCA: principal component analysis
