# Supplementary figures and images for: Use of Untargeted Liquid Chromatography–Mass Spectrometry Metabolome To Discriminate Italian Monovarietal Red Wines, Produced in Their Different Terroirs
Source: J Agric Food Chem. 2020 Apr 9;68(47):13353–66. doi: 10.1021/acs.jafc.0c00879 (PMC7997580; doi:10.1021/acs.jafc.0c00879)

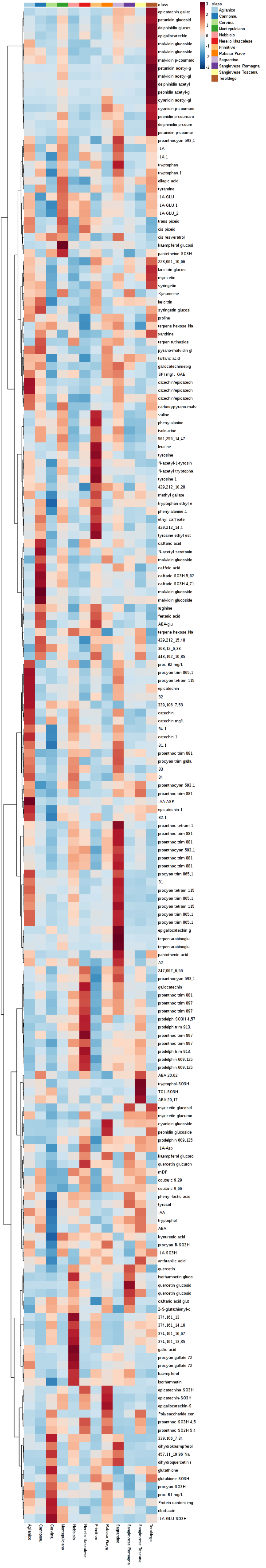

Supplement: Supplementary file 4 — jf0c00879_si_004.pdf [file jf0c00879_si_004.pdf]
